# Vascular reconstructions in living donor kidney transplantation: a single-center experience over the last 17 years

**DOI:** 10.3389/frtra.2024.1488277

**Published:** 2024-12-06

**Authors:** Nadina Roth, Manfred Kalteis, Axel Krause, Christiane Sophie Rösch, Jürgen Huber, Wolfgang Enkner, Maria Haller, Daniel Cejka, Reinhold Függer, Matthias Biebl

**Affiliations:** ^1^ Surgical Department for General, Visceral, Thoracic and Transplant Surgery, Ordensklinikum Linz Elisabethinen, Linz, Austria; ^2^ Faculty of Medicine, Johannes Kepler University Linz, Linz, Austria; ^3^ Department of Nephrology, Hypertension, Transplant Medicine, Rheumatology, Geriatrics Ordensklinikum Linz Elisabethinen, Linz, Austria

**Keywords:** living kidney donation, multiple arteries, arterial vascular reconstructions, transplant program, single-center follow-up

## Abstract

**Introduction:**

In living donor kidney transplantation (LDKT), vascular anastomosis is more difficult due to missing arterial patches and shorter renal veins. The surgical challenge is even more demanding in kidneys with multiple arteries. Although renal transplantation is feasible in most cases of complex donor vascular anatomy and similar results compared with standard LDKT are reported, the discussion on potentially increased complication rates and graft function continues. This prompted us to review our results of LDKT with multiple renal artery (MRA) grafts with a special concentration on complications and long-term function.

**Patients and methods:**

We reviewed the records of all LDKT in our center from the beginning of the program in 2005 until 2022 for arterial vascular reconstructions. The cohort was divided into two groups: transplantation with vascular reconstruction (VR) and standard transplantation. These groups were compared for operative parameters and short- and long-term results.

**Results:**

From 2005 to 2022, 211 LDKT were completed in our unit. In 32 (15.2%), a VR was performed, including single ostium side-to-side anastomosis, end-to-side anastomosis, patch reconstruction, and vein interposition. There was no significant difference in operative time (169 min vs. 180 min; *p* = 0.118) and time for anastomosis (28 min vs. 26 min; *p* = 0.59) between both groups. Postoperative complications (5.7% vs. 7.4%; *p* = 0.72) were not significantly different. During the follow-up period (110 months, range 10–204), the risk of organ loss was comparable after VR (13.625% vs. 11.56% *p* = 0.69).

**Conclusion:**

In LDKT, arterial vascular reconstructions for kidneys with MRA provide similar results compared to grafts with a single renal artery (SRA). Short- and long-term results are comparable with standard procedures.

## Introduction

Living donor kidney transplantation (LDKT) is an excellent treatment option for patients with end-stage renal disease. The advantages of living donation compared with kidney transplantation utilizing organs from deceased donors are short waiting times, the possibility of preemptive transplantation, planning the procedure electively, and, not least, better graft survival in general.

This has led to an increase in LDKT. Although LDKT is routinely performed in many transplant centers, removal and implantation are appraised as demanding. Among other issues, the implantation of kidneys with multiple renal arteries (MRA) is rated as a technical challenge, and clinical practice in accepting them for LDKT is not consistent amongst transplant centers.

Generally, the use of kidneys with MRA is a central issue of strategic considerations regarding organ acceptance and surgical technique. In LDKT, this topic is of utmost importance due to the expectation of superior graft function and minimized complication rates compared to transplantation utilizing organs from deceased donors. In an analysis of all laparoscopic donor nephrectomies in 10 years, no significant differences between complication rates and graft survival at 1 and 5 years were observed between grafts with MRA and SRA (1). However, data reported for living donor transplantation with MRA grafts are not homogenous and reflect interinstitutional differences. The spectrum includes comparable results, but also longer operation time and differing graft survival for diverse reconstruction techniques (2–4). In a meta-analysis including 23 studies, each comprising more than 50 patients with MRA and comparable data for grafts with a single renal artery (SRA), higher rates of postoperative complications and an increased risk for delayed graft function, but no difference with respect to long-term graft function and patient survival were reported (5).

These heterogenous data prompted us to retrospectively analyze the results with MRA grafts in LDKT in our center, concentrating especially on perioperative complication rates and graft survival.

## Patients and methods

### Patients and data collection

A total of 211 adult LDKT were performed between 1 January 2005 and 31 December 2022 at the OKL Elisabethinen Linz, Austria. All donors and recipients were enclosed in the routine preoperative evaluation program for LDKT. The vascular renal anatomy of donors was evaluated by magnet resonance angiography.

In 38 LDKT (18%), more than one artery was found; in 32 LDKT (15.2%), a vascular reconstruction (VR) was performed, including single ostium side-to-side anastomosis, end-to-side anastomosis, patch reconstruction, and vein interposition. In 6 of 38 donors, a second renal artery was ligated before implantation due to an estimated diameter of the lumen below 2 mm.

For analysis, the cohort was divided into two groups, transplantation with VR and standard transplantation. These groups were compared for the time of operation and anastomosis, warm and cold ischemic time, perioperative vascular and urological complications, significant bleeding, median creatinine serum levels, and graft and patient survival at 1, 5, and 10 years of follow-up. Patient characteristics, number of previous transplantations, ABO incompatibility (ABOi), and relation status of donors and recipients are summarized in Table 1.

**Table 1:** Study characteristics.

Study characteristics	211 recipients/211 donors
Mean donor age	54a (37–75 range)
Mean recipient age	44a (18–66 range)
Mean BMI donors	26.2 (19.7–34.4 range)
Mean BMI recipients	24.1 (19.9–33.3range)
Prior kidney transplantations	3 (1.4%)
ABOi transplantations	44 (20.85%)
Kreatinine preoperative donors	0.82 mg/dl (0.53–1.09)
Kreatinine postoperative donors 10a follow-up	1.18 mg/dl (0.71–1.57)
Related	55.8%
Related female donors	60%
Non-related female donors	71.6%
Male recipients	69.5%
Kreatinine recipients 10a follow-up	1.21 mg/dl (0.89–1.78)

ABOi, ABO incompatibility.

Information on the donation side and the surgical technique for donation is presented in Tables 2 and 3.

**Table 2:** Number of arteries in donors and time of reconstructions.

Donors	*n* = 211
Single renal artery	173 (84.8%)
Two renal arteries	35 (16.2%)
Three renal arteries	3 (1.4%)
Retroaortal veins	3 (1.4%)
Complex reconstructions	32 (15.2%)
Operation time with reconstruction	180 min
Operation time without reconstruction	169 min
Time of anastomosis with reconstruction	28 min
Time of anastomosis without reconstruction	26 min

**Table 3:** Type of operation in donors.

Type of donor procedure	*n* = 211
Left laparoscopic kidney donation SRA	142 (67.4%)
Right open kidney donation SRA	31 (14.7%)
Left laparoscopic kidney donation MRA	28 (13.2%)
% Right open kidney donation MRA	10 (4.7%)
Number of MR angiography preoperative	100%

MRA, multiple renal artery; SRA, single renal artery.

In the case of comparable function and regular anatomy, the left kidney was chosen for donation. A difference of up to 10% in split renal function was estimated as comparable. With respect to differences in the number of renal arteries, the kidney with fewer arteries was designated for removal given a comparable function. A retroaortal renal vein diagnosed in magnet resonance angiography was not rated as a contraindication for left kidney donation. The follow-up period was 110 months in the mean (range, 10–204 months).

### Immunosuppression regime

Our standard immunosuppression regimen is a combination of prednisolone, tacrolimus, and mycophenolate mofetil following an induction regimen with basiliximab or belatacept.

### Surgical technique

All laparoscopic living donor nephrectomies were performed by an intraperitoneal approach, with four trocars placed pararectal. The graft was removed with an endobag via a Pfannenstiel incision. Open donor nephrectomy was used to harvest the right kidneys by an oblique incision in the upper right quadrant. Vessels were secured by clamps and sutures, in the laparoscopic technique by vascular staplers. Surgical devices used for dissection and coagulation were sealing instruments and caustic ticks.

For implantation, a standardized anastomotic end-to-side technique was applied, with polypropylene 5-0, double-armed for the venous anastomosis and 6-0 for the arterial anastomosis. For arterial reconstruction of small renal arteries, polypropylene 7-0 was used. Implantation of the ureter was done by an ureterocystostomy in antireflux technique by Gregoire with a short double-J ureteral stent. The implantation of the ureter was performed with 6.0 absorbable monofilament double-armed sutures. The detrusor muscle was closed over the anastomosis with 5.0 absorbable sutures as an antireflux measure.

### Statistical analysis

Data collection was carried out by one author, and the essential variables were collected in an electronic database (Microsoft Excel). The categorical variables were reported as frequencies and proportions, and continuous data were reported as median, minimum, maximum, and range. For metric values with normal distribution, the standard *t*-test and Fisher’s exact test were used.

## Results

Overall, there were 173 (82%) implanted grafts with a SRA, and 35 (15.2%) with two and three (1.4%) with three arteries (Table 2). In total in 32 of 38 patients (15.2%), complex arterial reconstructions on the back table prior to implantation were performed. In 6 of the 38 patients, an upper pole artery was not reconstructed because of too small lumina. Analyzing the distribution of left and right kidneys used for transplantation in the MRA and SRA groups, we found, that the donation side of grafts with MRA was left in 28 (13.2%) and right in 10 (4.7%) donors. In the SRA group, there were 142 (67.4%) left and 31 (14.7%) right kidneys donated. The distribution of the donation side was not significantly different in the MRA and SRA groups (*p* = 0.26, Fisher’s exact test; Table 3).

The vascular anatomy of transplanted kidneys and details on operative and anastomotic times are shown in Table 4. In all donors with MRA, their specific anatomy had been diagnosed during the preoperative workup.

**Table 4:** Different complex arterial reconstructions.

Complex arterial reconstructions	32 (15.2%)
Single ostium side-to-side anastomosis	9
End-to-side anastomosis	12
Side-to-side and end-to-side anastomosis	3
Patch reconstruction	1
Vein interposition (V. saphena magna)	1
Two arteries single reconstructed	2
Disobliteration of A. iliaca externa and overcross bypass	1
Arteriotomy because of early branch in a short renal artery	3

There was no significant difference in operative time (180 min vs. 169 min; *p* = 0.118) and mean time for anastomosis (28 min vs. 26 min; *p* = 0.59) between recipients with MRA and SRA, respectively. For reconstruction and implantation of MRA kidneys, four surgical techniques were applied, according to particular anatomic situations (Tables 2, 4).

**Figure 1:**
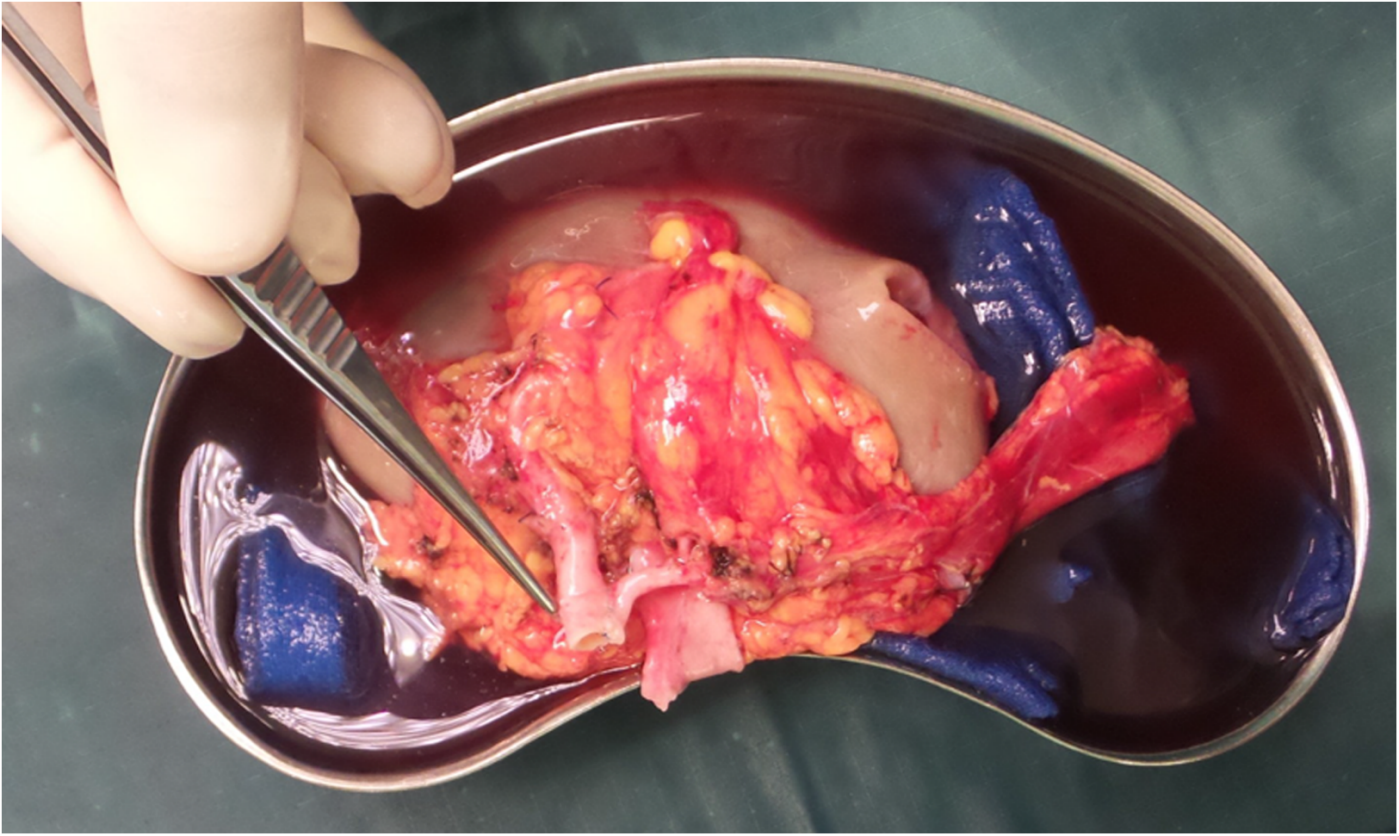
Side-to-side anastomosis with single ostium.

**Figure 2:**
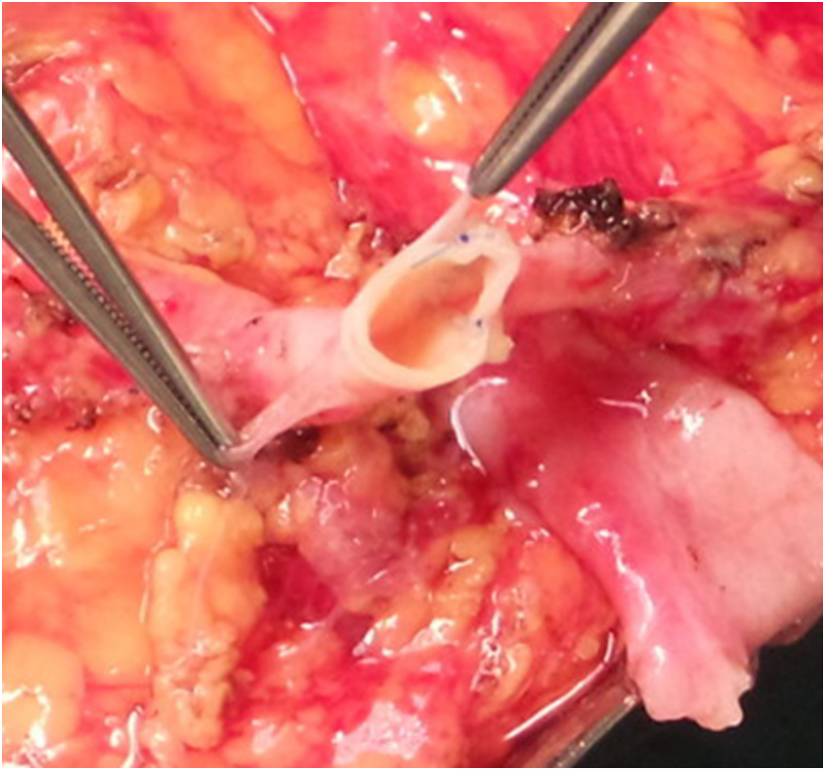
End-to-side anastomosis.

**Figure 3:**
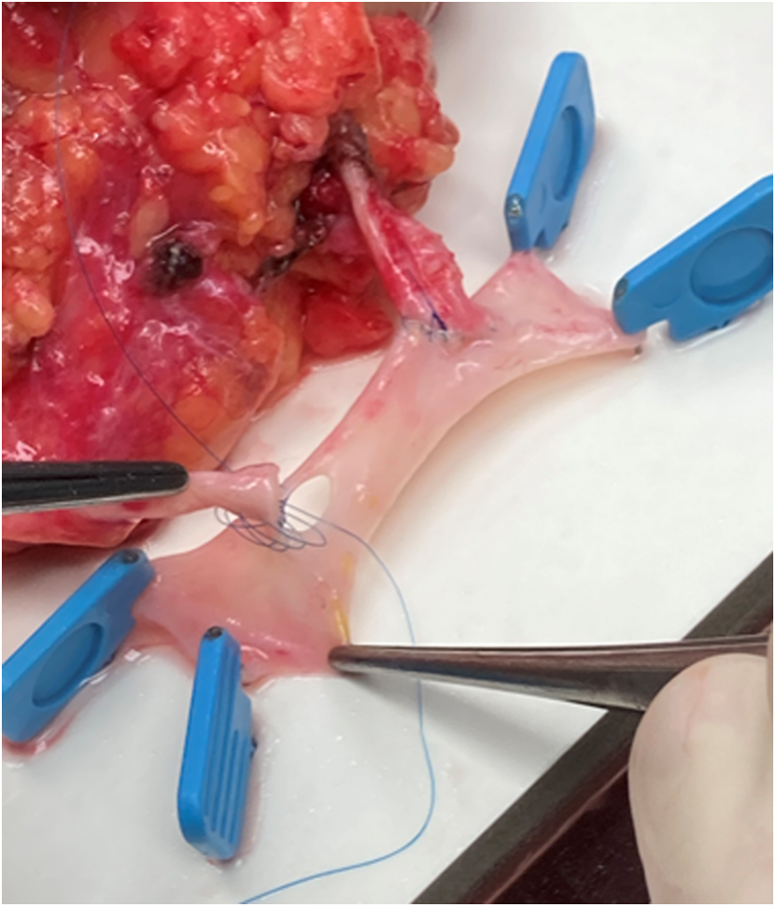
Vein interposition (V. saphena magna).

**Figure 4:**
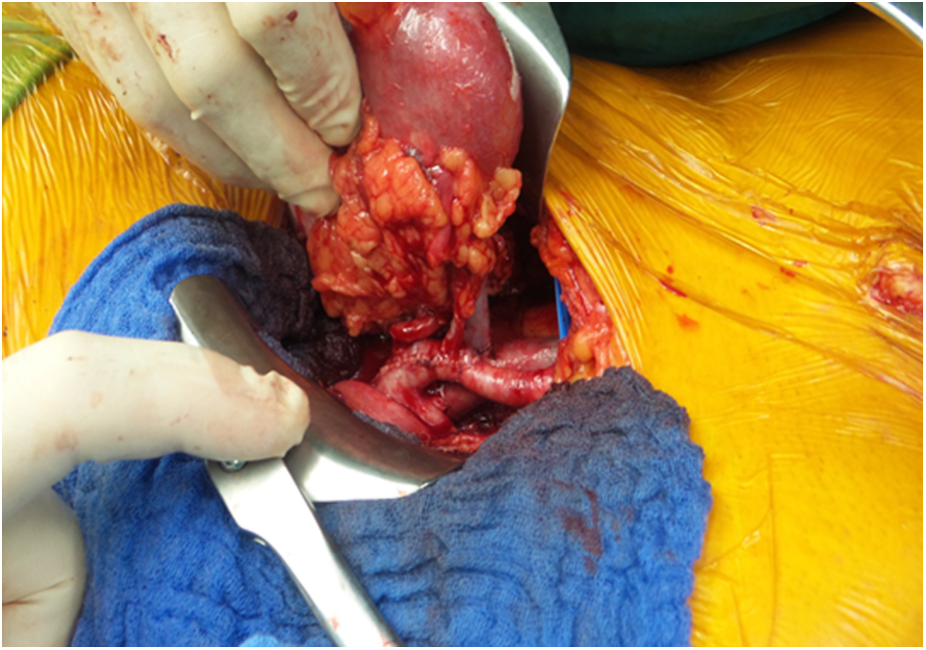
Two arteries single reconstructed.

We documented nine single ostium side-to-side anastomoses (Figure 1) and twelve end-to-side anastomoses (Figure 2). In three patients, grafts with three renal arteries were implanted, using a combination of side-to-side and end-to-side anastomosis with patch reconstruction and vein interposition (Figure 3). In two patients, kidneys with two distant renal arteries were implanted choosing two separated arterial anastomoses (Figure 4).

Aside from arterial reconstructions in patients with MRA, vascular surgical interventions were performed in four patients with SRA. In three patients, the first arterial branch of the renal artery originated close to the transection line of the renal artery. An arteriotomy from the transection line into this branch was performed, to include the side branch into the arterial anastomosis with the external iliac artery of the recipient. In one patient, a thrombendarterectomy of the external iliac artery distal of the arterial anastomosis and a femorofemoral overcross bypass were performed due to ischemia of the leg following reperfusion of the graft.

Retroaortal veins were found in three patients (1.4%).

Postoperative complications are summarized in Table 5.

**Table 5:** Vascular and urological complications.

Follow-up total *n* = 211	173 SRA/38 MRA		
Vascular and urological complications	173 SRA	32 MRA	6 MRA with upper pole arteries not reconstructed
	Left/right	Left/right	Left/right
Venous thrombosis	1/0	0/1	0
Arterial thrombosis	1/0	0/1	0
Cellular acute rejection ABOi	2/0	0/1	0
Urinary leaks	1/1	0/0	0
Ureteral necrosis stricture	1/1	0/0	0
Lymphocele	1/0	0/2	0
Postoperative complications/reoperations	7.4%	5.7%	-

MRA, multiple renal artery; SRA, single renal artery.

In three patients in MRA (*n* = 3/32), a graft loss emerged due to postoperative complications within 30 days after transplantation. All of them had received a right kidney. There was one renal vein thrombosis with consecutive significant bleeding of the kidney that resulted in organ loss five days after transplantation in an ABOi transplantation (Table 6) with histologically proven acute cellular rejection. In a second patient, after implantation of a graft with three renal arteries and reconstruction by vein interposition, ischemia and non-perfusion of the transplanted kidney due to occlusion of the arterial reconstruction were diagnosed on postoperative day 5. In the third patient, a small SRA was cut unintentionally and without notice during preparation with a sealing instrument. This was realized at the time of graft removal, resulting in a warm ischemic time of ∼10 min. After perfusion and reconstruction by vein interposition and consecutive implantation in the recipient, the graft had to be explanted due to non-perfusion in the further course.

**Table 6:** Complications in ABOi transplantation.

3 ABOi acute cellular rejections/ABOi graft loss	1	2	3
Donor blood type	A+	B+	A+
Recipient blood type	O+	A+	O+
Titer on the day of surgery	1:2	1:4	1:2
Titer one day after surgery	1:4	1:8	1:4

In SRA transplantation, we lost four left kidneys (*n* = 4/173, 2.3%) within 30 days. The issues were one venous thrombosis, one arterial thrombosis with significant bleeding afterward, and two acute cellular rejections in the ABOi transplant program which are histologically proven (Table 6).

Overall the risk of organ loss was not significantly different after VR compared with transplantations of SRA kidneys in the first year with 6.92% in SRA and 7.89% in MRA. The 10-year follow-up showed 11.56% graft loss in SRA vs. 13.625% in MRA, *p* = 0.69 (Table 7).

**Table 7:** Follow up graft loss and creatinine median.

Follow-up total *n* = 211	173 SRA	32 MRA	6 MRA with upper pole arteries not reconstructed	*p*-value
Graft loss 1a (100% follow-up)	6.92%	7.89%	0	n.s.
Graft loss 110 months, (mean range 10–204 months) *n* = 197, 93.4% follow-up	11.56%	13.625%	0	0.69
Creatinine median (mg/dl) (follow-up *n* = 197, 93.4%)				
1a follow-up *n* = 18	1.18	1.17	1.17	
5a follow-up *n* = 46	1.17	1.14	1.14	
10a follow-up *n* = 80	1.16	1.15	1.15	
15a follow-up *n* = 53	1.14	-	-	

n.s., not significant; MRA, multiple renal artery; SRA, single renal artery; ABOi, ABO incompatibility.

Other postoperative complication rates of transplantations with MRA and SRA kidneys were comparable (5.7% vs. 7.4%; *p* = 0.72). No complications were observed in the group of six patients with a ligated small upper pole artery without reconstruction.

Creatinine serum levels of kidneys with arterial reconstruction revealed no significant difference compared to SRA kidneys in the long-term follow-up.

## Discussion

Living donated kidney transplantation is increasingly utilized in patients awaiting transplantation. Major arguments are long waiting lists due to the shortage of organs from deceased donors, the possibility of preemptive transplantation, the change to an electively planned procedure, and superior graft survival in long-term follow-up. Hence, functional and anatomical selection criteria for living donation are even more the focus of pretransplant considerations. It is consensus to leave the kidney with superior function with the donor and, in case of comparable renal function, to choose kidneys with regular anatomy for removal. However, the literature is not uniform with respect to the preferred side of removal, in view of the shorter right renal vein and the implantation of grafts with MRA. There is an increasingly frequent situation in which a decision upon eligibility of kidneys with upper, more rarely lower, pole arteries or multiple arteries for transplantation has to be taken. MRA are reported unilaterally in 23%–25% and bilaterally in 6%–10% (6). This corresponds with our series of 18% unilateral and 7.4% bilateral MRA and underlines the obligatory depiction of vascular anatomy by preoperative magnetic resonance angiography.

Studies analyzing the results of transplantations of kidneys with MRA are mostly case series with limited patient numbers and restricted comparability. Although conclusions from these reports are not uniform and recommendations lack higher scientific evidence, there is a trend to abandon restrictions for the transplantation of grafts with MRA. Currently, the evidence of living donation kidney transplantation using organs with MRA is best summarized in a recent meta-analysis by Lim et al., comprising 14 cohort studies with Kaplan–Meier curves for graft survival and 9 studies for overall survival in LDKT with SRA and MRA organs (7). The authors found no significant difference in GS and OS and concluded that MRA should not be a selection factor in evaluating suitable kidneys for transplantation. Another systematic review was published by Zordrager et al. in 2016 (5). In this study, grafts of any kind of donation were analyzed for a possible difference between MRA and SRA organs regarding the outcome. A total of 23 studies with 18,289 patients were included. They found higher complication rates, an increased incidence of delayed graft function, and significantly lower 1-year graft survival (93.2% vs. 94.5%, OR 0.819, *p* = 0.022) in MRA compared with SRA transplantations. However, long-term outcomes were not markedly different with 5-year graft survival of 81.4% vs. 81.6% and 5-year patient survival of 89.6% vs. 87.0% in MRA and SRA groups, respectively. Not surprisingly, these reviews and published cohort studies concentrate on the incidence of complications, especially vascular and urological, and long-term graft survival, when comparing transplantations of MRA and SRA kidneys. In a prospectively documented survey of the consequences of MRA kidneys in LDKT, Kok et al. reported a higher rate of ureteral complications in patients with lower pol arteries (3). This was a specific finding for accessory lower pol arteries, because neither MRA nor VR were of influence on ureteral complications in general.

Other institutional series report no significant differences in vascular and urologic complication rates and graft survival, when comparing SRA and MRA kidneys in LDKT (1, 8). Chedid et al. reported vascular complications in 1.1% vs. 2.4% (*p* = 0.17), urologic complications in 3.1% vs. 2.9% (*p* = 0.47), and 5-year graft survival of 83.5% vs. 82.6% (*p* = 0.82) for SRA and MRA kidneys in LDKT (1). In our series, we found similar overall complication rates (5.7% vs. 7.4%). However, three patients of the MRA group lost their graft within 30 days postoperatively. Detailed analysis revealed that only one graft loss was caused by occlusion of the arterial reconstruction in a donor kidney with three renal arteries on postoperative day 5. The other early graft losses were not attributable to the use of MRA kidneys for transplantation. One patient developed a thrombosis of the renal vein after transplantation of a right kidney, and another patient suffered from an iatrogenic injury of a renal artery during open removal of a right kidney with two renal arteries and lost the graft due to prolonged warm ischemic time and primary non-function. Despite this high rate of early graft losses with 6.9% in SRA vs. 7.9% in MRA in the first year of our cohort, long-term function at 10 years was comparable in our MRA and SRA groups. Similarly, graft losses at a median long-term follow-up of 110 months were not different between MRA and SRA groups with 13.6% vs. 11.6%, respectively, and at a comparable range with published data. The long-term follow-up enabling a comparison of kidney function between SRA and MRA grafts is a strength of our study.

The incidence of venous and arterial thrombotic occlusions in our total cohort was 1.9% (*n* = 4/211) which corresponds to 2.4% reported in the series of Sagban et al. (9).

We did not observe a difference in ureteral complications between SRA and MRA transplantations. In accordance with the meta-analysis of Zorgdrager (5), the analysis of ureteral complications in transplantations with MRA grafts may require a more differentiated approach concentrating on lower pole arteries. We did not observe a lower pole artery among our MRA kidneys. In our series, all living donor kidney recipients got double-J stents to prevent mechanical ureteral complications. This is a routine measure in our deceased donors’ kidney transplantation program as well as in many transplant centers.

Some authors argue that every vascular pathology can be reconstructed in a highly specialized transplant at the center (8, 9). Indeed, there are reports on different surgical techniques for arterial reconstruction in MRA grafts. The most common technique is side-to-side anastomosis, and others describe end-to-side anastomosis or reconstruction with a patch (2, 4, 10). Although there are differences in graft function described, there is no superior standard for arterial reconstruction described. Tabbara et al. described 18 different reconstruction techniques in 70 grafts with MRA, which underlines the absence of an evidence-based standard (2). In our series, end-to-side, side-to-side, vein interposition, and separate arterial anastomosis with the iliac artery of the recipient were performed. We did not observe clinically relevant differences between the techniques.

Another issue in the discussion of using MRA kidneys for LDKT is, whether a left kidney with multiple arteries or a right kidney with a singular renal artery should be chosen for removal. While the right renal vein is short in laparoscopic donor nephrectomy and an increased risk for venous thrombosis is assumed, the left kidney provides the advantage of a longer vein for anastomosis. Therefore, left donor nephrectomy is preferred in most transplant centers. In a comparison of 34 left MRA and 39 right SRA grafts, three patients with graft loss due to venous thrombosis after transplantation of right kidneys were reported. The authors conclude that left MRA kidneys could be a better option than right kidneys with a SRA (11).

In our series, six patients had upper pole arteries with an estimated diameter below 2 mm that were primarily ligated. In this group, we had no higher incidence of vascular or urological complications and good functional results. Upper pole arteries of this seize vascularize 5%–10% of the kidney (12). There is a controversial discussion about whether such small pole arteries should be reconstructed or ligated (5, 13). Iwami et al. reported that ligation of an upper pole artery with a diameter of 2 mm leads to an estimated parenchyma loss of 8% and recommend reconstruction above this cutoff.

A meta-analysis found a prolongation of the warm ischemic time in transplantations of MRA kidneys leading to increased delayed graft function (5). Excluding the patient suffering from the iatrogenic injury of the main renal artery and consecutive prolonged warm ischemic time, warm and cold ischemic times did not influence the functional outcome in our series. The arterial reconstruction was performed with the graft adequately perfused and cooled. While the finding of MRA was known because of preoperative magnetic resonance angiography, the surgical team was prepared. VR was performed on the back table on the perfused and cooled graft (5, 9). Cold ischemic time was kept short by two surgical teams for removal and implantation, working overlapping in two operating theaters.

Our study is limited by several issues. The most important item is the retrospective study design and the limited prospective documentation. The long study period may increase this shortcoming, although this allows a long follow-up. The number of patients in this cohort is relatively small, albeit within the range of other single institutional series.

In conclusion, LDKT using grafts with MRA is increasingly performed. This is a consequence of efforts to enlarge suitable donors and is supported by data demonstrating comparable long-term results. However, there is still a controversy about whether arterial reconstructions are associated with a higher postoperative complication rate, and the question of whether a short right renal vein or a left kidney with multiple arteries is the more appropriate organ for LDKT remains open. Finally, the decision to use MRA grafts remains an individual decision based on donor and recipient parameters and the experience of the transplant center.

## Data Availability

The original contributions presented in the study are included in the article/Supplementary Material; further inquiries can be directed to the corresponding author.
